# Mast Cell Activation Disorders

**DOI:** 10.3390/medicina57020124

**Published:** 2021-01-30

**Authors:** Arianna Giannetti, Emanuele Filice, Carlo Caffarelli, Giampaolo Ricci, Andrea Pession

**Affiliations:** 1 Pediatric Unit, IRCCS Azienda Ospedaliero-Univerisitaria di Bologna, 40138 Bologna, Italy; ariannagiannetti25@gmail.com (A.G.); eman.filice@gmail.com (E.F.); giampaolo.ricci@unibo.it (G.R.); 2 Clinica Pediatrica, Department of Medicine and Surgery, University of Parma, 43126 Parma, Italy; carlo.caffarelli@unipr.it

**Keywords:** mast cell, tryptase, mastocytosis, hereditary alpha-tryptasemia, C-Kit, N-methylhistamine, 1,4-methylhistamine, urinary leukotriene E4, 9 alpha,11 beta- prostaglandin F2α

## Abstract

*Background and Objectives:* Mast cell disorders comprise a wide spectrum of syndromes caused by mast cells’ degranulation with acute or chronic clinical manifestations. *Materials and Methods:* In this review article we reviewed the latest findings in scientific papers about mast cell disorders with a particular focus on mast cell activation syndrome and mastocytosis in pediatric age. *Results:* Patients with mast cell activation syndrome have a normal number of mast cells that are hyperreactive upon stimulation of various triggers. We tried to emphasize the diagnostic criteria, differential diagnosis, and therapeutic strategies. Another primary mast cell disorder is mastocytosis, a condition with a long-known disease, in which patients have an increased number of mast cells that accumulate in different regions of the body with different clinical evolution in pediatric age. *Conclusions:* Mast cell activation syndrome overlaps with different clinical entities. No consensus was found on biomarkers and no clearly resolutive treatment is available. Therefore, a more detailed knowledge of this syndrome is of fundamental importance for a correct diagnosis and effective therapy.

## 1. Introduction

Primary mast cell disorders include monoclonal mast cell activation syndrome (MCAS) and mastocytosis. MCAS is a recently recognized entity, belonging to the wide spectrum of syndromes provoked by mast cells’ (MCs) degranulation.

Because of the great heterogeneity of its clinical pattern, it has been underdiagnosed for a long time, requiring a huge effort in standardizing its definition and management. Furthermore, misdiagnosis is common since it is often not recognized as such and the number of reported cases is like the tip of an iceberg [1].

Mastocytosis, on the contrary, defines a long-known group of disorders whose main feature is represented by the accumulation of MCs in different body regions.

## 2. Background on Pathophysiology of Mast Cell Degranulation

Much has been written about MC function. MCs represent the effector cells of allergy. Their activation is classically induced by the bounding of allergens to the high-affinity Fc epsilon receptor 1 (FCeRI), but also by a wide range of other mediators such as complement anaphylotoxins, physical triggers, drugs, physical stimuli, cytokines, and more [2].

MCs are located inside the connective tissue of vascularized organs, in mucosal tissues (respiratory and intestinal, exerting a “sentinel” function), and mostly in the skin. They contain many cytoplasmic granules containing prestored mediators, including histamine and tryptase, that are massively released in the blood stream after MCs are activated, leading to clinical manifestations.

These cells produce not only pro-inflammatory mediators and cytokines, including histamine, cysteinyl leukotrienes, and prostaglandins, but also certain proteoglycans such as heparin and various proteases [2,3,4]. Tryptases are serine proteases that are preferentially produced and stored in MCs [5,6] and account for >20% of the total protein content in tissue MCs [7]. While mature tryptases are mostly stored in the metachromatic granules, the precursor forms (pro-tryptases) are released spontaneously and continuously from resting MCs [8,9]. Based on a stable release rate of tryptases, the resulting basal serum tryptase level is extremely constant in healthy individuals. Only during and soon after a severe anaphylactic event where MCs release large amounts of tryptase, the serum tryptase level increases significantly over the individual’s baseline [10,11,12,13,14,15]. Subsequently, the tryptase level returns to baseline, in several hours (up to 24 h), providing a consistent diagnostic window of about 2–4 h for laboratory tests [16] and if other chemical mediators, like histamine, prostaglandin D2, and heparin, may also provide as indicators. In daily practice, tryptase is considered the reliable biomarker of severe systemic MC activation [10,11,12,13,14,15,16]. Tryptase is also released by activated basophils, but MCs contain 500-fold higher levels of basophils, so that tryptase results in being somewhat specific for their function.

More than 90% of patients with mastocytosis have a somatic gain-of-function mutation in the KIT receptor tyrosine kinase, primarily an aspartic acid-to-valine substitution (D816V), which results in enhanced survival and cell autonomous growth of neoplastic mast cells (MCs). Under normal physiological conditions, MCs are strongly regulated by the availability of KIT ligand Stem Cell Factor (SCF). Otherwise, under pathological conditions, uncontrolled proliferation and enhanced survival of MCs can contribute to the disease pathogenesis [17].

## 3. Definition of MCAS

According to 2012 diagnostic criteria by Valent et al. [18] and the subsequent 2019 update [19], MCAS can be diagnosed by the simultaneous presence of:

- typical clinical signs and symptoms of severe recurrent acute systemic (involving at least two organ systems) MC activation.
- laboratory-confirmed MCs’ involvement, defined by increase in serum total tryptase, histamine or its metabolites, or prostaglandin D2 or its urinary metabolites compared to a baseline level.
- favorable response to drugs with MC-stabilizing agents or acting against MC-derived mediators.

The typical signs and symptoms associated with MC activation are flushing and hypotension (for a 95% of consensus level among the authors), pruritus, nasal congestion, nasal pruritus, headache and diarrhea (90% consensus level), urticaria and throat swelling (85% consensus level), angioedema (75%), and wheezing (70%). None of them is specific to MCAS, even if rash, urticaria, and wheezing [20] are more typical of mast cell activation. MCAS should be suspected when complaints are episodic, reiterated, and unexplained.

In order to define a systemic involvement, at least two organ systems must be affected.

Serum total tryptase is considered the preferred biomarker for diagnosing MCAS. It requires to be analyzed through a fluoroimmune enzyme assay and, ideally, the blood sample should be promptly collected after onset of symptoms because of the short half-life of the enzyme. In order to define a significative increase in tryptase levels above the baseline, the level of the enzyme should be higher than 20% plus 2 ng/mL [21]. When a baseline level prior to the onset of acute phase is not available, it can be detected at least 24 h following acute phase’s complete resolution.

No consensus has been reached on the diagnostic role of different biomarkers, though the urinary histamine metabolites N-methylhistamine and 1,4-methylhistamine, urinary leukotriene E4 (LTE4), and 9 alpha,11 beta-prostaglandin F2α (9a-11b-PGF2) appear to be promising.

Urinary level of 9a-11b-PGF2 24-h is higher within 3 h from acute episode when compared to controls and appears to be suggestive of idiopathic MCAS. Though more difficult to collect, 9a-11b-PGF2 concentration was found to be more sensitive than tryptase for MCAS and more specific for MC activation since it derives from prostaglandin D2 (PGD2), a molecule that is not produced by basophils [22].

MCAS can be classified into three different subtypes (Table 1).

The presence of a clonal line of MCs, which often express CD25, carrying a mutation in c-kit protooncogene defines “primary MCAS” or “clonal MCAS”. C-Kit gene encodes KIT, a type III transmembrane tyrosine kinase with an extracellular domain activated by SCF [23].

When c-kit gene is mutated, the activation of its product KIT becomes SCF-independent and constitutive.

When an underlying disease such as allergy or autoimmune disorders is diagnosed, MCAS is defined as “secondary”.

Finally, patients who do not qualify for either primary or secondary MCAS are diagnosed with “idiopathic MCAS”.

MCAS overlaps with different clinical entities. According to a multicenter European research from 2019 on 2083 patients suffering from functional gastrointestinal disorders (FGID), 85% of patients presented a clinical overlap between the two entities. MCAs was most likely overdiagnosed in this study because of the adopted diagnostic definition, which did not fulfill the 2012 consensus. Consequently, the conclusions drawn from the authors are to be carefully analyzed. Nevertheless, interestingly, FGID appear to be characterized by mast cell involvement, testified by both biopsy findings and urinalysis [24].

Weiler [25] recently stressed that probably too many medical conditions are associated to MCAS, often leading to misdiagnosis and possibly disproportionate therapies with no benefit for either the physician or the patient.

## 4. Definition of Mastocytosis

Mastocytosis is defined by an abnormal clonal MCs’ expansion, leading to their accumulation in many body regions, mainly skin and bone marrow [26].

Its prevalence rate is estimated at 9 cases per 100,000 persons in the USA [27] and 13 cases per 100,000 persons in the Dutch region, aged over 15 years [28]. About 35 out of 100 cases of cutaneous mastocytosis and 1.8 out of 100 cases of systemic mastocytosis occur in children [29].

Despite a long history, started in 1869 with the first description of Cutaneous Mastocitosys [30], it was only in 2016 that World Health Organization (WHO) finally recognized mastocytosis as a distinct disease category no longer belonging to myeloproliferative neoplasms [31].

Under the category of Mastocytosis, a wide range of clinical forms is included, ranging from the most benign and, luckily, most common “Indolent Systemic Mastocytosis” to the rarer but potentially lethal “Mast-Cell Leukemia” and “Mast Cell Sarcoma” [2].

WHO puts a huge effort to organize the heterogeneity of mastocytosis into different nosological categories (Table 2).

Cutaneous mastocytosis (CM) is the most common form in the pediatric population, consisting of a local accumulation of MCs limited to the skin and, in its turn, has three different forms:

- Maculopapular CM (MPCM), also known as urticaria pigmentosa, with its two variants, monomorphic and polymorphic [32]. Clinically, it is recognizable for its small, monomorphic, red-brown macules/papules ,typically on the trunk, sparing face and extremities [33].
- Diffuse CM is uncommon, accounting for no more than 2% of all forms of CM, and consists of an infiltration of MCs involving the entire skin, often with a neonatal onset [34].
- Solitary/localized cutaneous mastocytoma, accounting for up to 15% of all pediatric forms of CM, is characterized by a limited accumulation in a specific skin site, typically post-traumatic (e.g., in the sites of vaccination) probably following the releas of SCF, nerve growth factor, and interleukin-5 [27].

CM is to be suspected when Darier sign is present, namely, the induction of localized urticaria and erythema after rubbing, scratching, or stroking affected skin because of the release of MC mediators [35].

Definite diagnosis requires a skin biopsy with immunohistochemical staining for tryptase and KIT.

In most cases, CM develops with a benign course and often regresses by puberty. Recent data showed that localized mastocytomas tend to regress at a rate of 10% per year (Gurnee), while MPCM presents a slower rate of 1.9% per year [32,36,37].

Systemic Mastocytosis (SM) is, on the contrary, characterized by a multi-organ involvement that requires a differential diagnosis from MCAS and usually involves the bone marrow (BM), spleen, liver, and gastrointestinal tract. Table 3 depicts the diagnostic criteria for SM.

Among the different forms of SM, indolent systemic mastocytosis (ISM) is, luckily, the most common. It is characterized by a stable or slowly progressing clinical course, often with a good prognosis [38].

Smoldering systemic mastocytosis (SSM) is a more invasive and rarer form, overlapping with myelodysplasia, caused by a huge involvement of bone marrow by MCs (>30%).

Systemic mastocytosis with an associated hematological neoplasm (SM-AHN) is, as its name suggests, a form of SM possibly accompanying all forms of hematological neoplasms. It accounts for up to 40% of all SM cases (second most frequent subvariant following ISM) [38].

Aggressive systemic mastocytosis (ASM) is a fearsome, relatively rare (12%) form of SM where the term “aggressive” refers to the entity of involvement of tissue infiltration, potentially leading to multi-organ dysfunction.

A special form of ASM is transforming ASM, a transitional state from ASM to Mast cell leukemia (MCL).

MCL is a rare, aggressive form with a poor prognosis, where immature forms of MCs are found both in peripheral blood and bone marrow.

Finally, mast cell sarcoma (MCS) is a poorly known (23 cases in the literature), destructive, neoplastic form in which neoplastic MCs are found often in bone tissue [39].

## 5. MCAS vs. Mastocytosis

Primary MCAS and Systemic Mastocytosis (SM) share a common genetic mutation since in both entities c-Kit shows a missense mutation (D816V). The mutation occurs in the vast majority of adult SM, regardless of the nosological category [31].

In pediatric mastocytosis, c-Kit activating mutations have been suggested to have a germinal origin with a wide number of different mutations, rather than just the D816V one, being found [31].

When in doubt between primary MCAS and SM, the clinical features need to be thoroughly evaluated: If criteria for diagnosing SM (Table 3) are not met, primary MCAS can be diagnosed.

Since both MCAS and mastocytosis share a massive activation of mast cells and consequent laboratory anomalies, Theoharides et al. recommended to perform a bone marrow biopsy in order to rule out a SM before safely diagnosing a MCAS [2].

The authors’ recommendation applies mainly to adult patients with typical skin lesions or unexplained hypotensive episodes and/or syncope and, secondly, to patients who also present unexplained osteoporosis, splenomegaly, or lymphadenopathy.

Pediatric patients, on the contrary, should not undergo bone marrow biopsy unless anomalies in spleen, liver, lymph nodes, and peripheral blood are present, because of the rarity of SM and of the lower frequency of V816F mutation in this age group [40].

## 6. Differential Diagnosis

To make things even more complicated, researchers recently found that up to 4% of general populations presents an autosomal dominant-inherited genetic feature, described as Hereditary Alpha-Tryptasemia (HAT) and consisting of an increase in the numbers of copies for one of the genes encoding for serum tryptase that results in increased baseline serum tryptase levels. Clinical manifestations are very variable, including atopy (94% of patients), gastroesophageal reflux disease (up to 100%), abdominal pain with diarrhea (87%), and cutaneous manifestations such as flushing, pruritus, and urticaria (up to 80%), often mimicking those of MC disorders [25]. Faced with these findings, Wieler [25] recommends to test for alpha-tryptase gene copy number in patients fulfilling all three MCAS criteria before diagnosing MCAS, in order to properly rule out the chance of a HAT.

Differential diagnoses include a huge number of clinical conditions potentially mimicking MCAS or mastocytosis, some sharing a pathogenesis involving MCs (such as allergic reactions, scombroid syndrome) and most characterized by completely different pathogenesis.

Table 4 (adapted from Weiler and Caimmi [25,41]) shows the most frequent.

Cardiovascular conditions, including acute myocardial infarction, myocarditis (relatively frequent in pediatric population), and hypovolemic shock, are easier to be ruled out because of the typical accompanying symptoms usually with a suggesting clinical history.

Skin disease such as atopic dermatitis or allergy contact dermatitis are excluded by clinical criteria.

A variety of endocrine diseases, including adrenal gland and thyroid dysfunction (both hypofunction and hyperfunction), can be ruled out by typical signs (goiter, excessive hair growth, striae rubrae, bronzing of skin) followed by laboratory findings.

Singular conditions, especially in pediatric patients (such as neurological or gastrointestinal diseases), may need specialist consultations.

## 7. Proposed Diagnostic Algorithm

**Figure 1:**
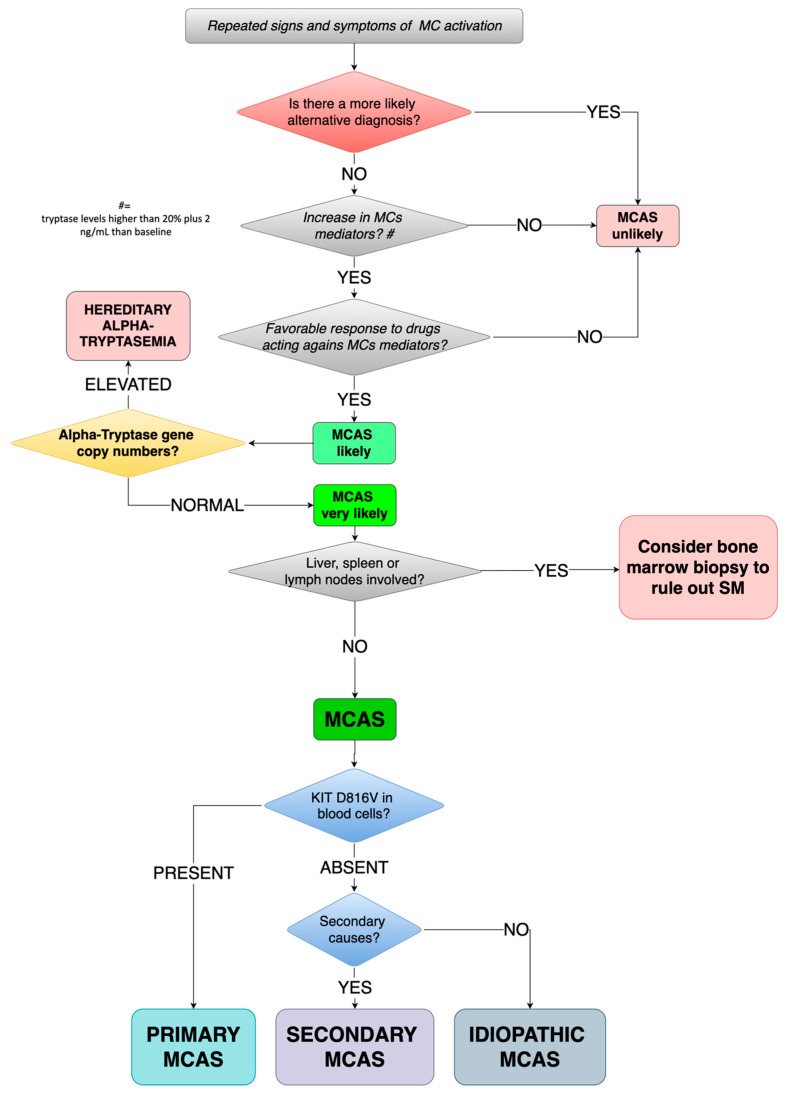
Proposed diagnostic algorithm for MCAS in children. MC(s): mast cell(s); MCAS: mast cell activation syndrome; SM: systemic mastocytosis.

In Figure 1 we have proposed a diagnostic algorithm of MCAS for children.

In patients suffering from repeated signs and symptoms of MC activation, we suggest testing for laboratory levels of mediators released by MCs, according to the availability of local laboratory, tryptase being the patient shows a significant most feasible. If both the previous conditions are satisfied and the favorable response to drugs acting against MC mediators (such as H1/H2-histamine receptor blockers, Leukotriene receptor blockers, or Mast cell stabilizers), MCAS is to be taken into account.

As previously stated, all three features need to be fulfilled at the same time.

Before proceeding with diagnosing MCAS (always keeping in mind that when hearing hoof beats, a horse should be suspected before zebras), clinicians should carefully run a re-evaluation of all possible differential diagnoses (Table 4).

If MCAS still remains the more likely diagnosis, Hereditary Alpha-Tryptasemia should be ruled out by testing for alpha-tryptase gene copy number: Increased numbers of copies are diagnostic for HAT while a normal number of copies should lead to diagnosis of MCAS.

At this stage, MCAS needs to be further characterized. The characterizing c-Kit mutation at D816V is to be searched in peripheral blood cells: When present, primary/clonal MCAS can be diagnosed. If D816V is absent, MCAS can be secondary or idiopathic. If a condition such as atopy, autoimmune diseases, bacterial infections, or adverse drug reaction is found, MCAS can be classified as secondary, otherwise as idiopathic. 

## 8. Treatment Options

### Management of MCAS and Mastocytosis

No resolutive treatment is available yet for MCAS, so its management, such as that of other MC disorders, needs to be multifactorial and ideally coordinated by comprehensive care centers with adequate experience.

The purpose of management is to minimize, as much as possible, the risk for recurrence of symptoms and, when symptoms are present, to efficiently treat them.

Firstly, Castells and Butterfield [42] recommend to adequately educate patients to avoid any kind of factor that may trigger degranulation of MCs (Table 5) and to use premedication regimens when about to undergo surgery, invasive procedures, or radiology procedures.

In avoidance of triggers, anesthesia requires a specific mention. A huge Spanish study from 2015 retrospectively evaluated the data of 726 anesthetic procedures performed in 501 patients (both pediatric and adults) suffering from mastocytosis. Authors found a higher frequency of perioperative anaphylaxis than in the general population but still recommended not to avoid anesthesia in patients with mastocytosis though managing them as high-risk patients, premedicating them with histamine receptor blockers and benzodiazepines 1 h before procedure to relieve stress [43].

Coltoff et al. recommend to prescribe epinephrine autoinjectors to all patients with known SM. This recommendation is explained by the tendency of patients with mastocytosis to develop more severe forms of anaphylactic reactions potentially leading to death [38].

Especially hymenoptera stings represent a very dangerous situation for patients. Studies have been demonstrating that they induce more severe systemic reactions than in patients who do not suffer from MC disorders. Hymenoptera stings are considered to be the most frequent risk factor for anaphylaxis in patients with MCAS or mastocytosis who are, consequently, eligible for specific venom immunotherapy (VIT) who appear to significantly reduce the risk for anaphylactic recurrence. VIT safety in patients suffering from MCs’ disorders appears to be safe and life-saving. To reduce both symptoms and adverse reactions, Omalizumab was tested and showed satisfactory results [44,45].

All patients suffering from MC disorders with anaphylactic reaction should be provided with two intramuscular epinephrine autoinjectors. The jab can be repeated no more than twice (1 to 5 min after the first administration) in case of massive MC activation.

Vaccination is a major concern in the pediatric population suffering from MCs’ disorders.

Immunization schedule in Europe and US begins very early in life and accompanies children for the first years of life.

Case reports have been describing a huge range of reactions to vaccination in this particular population, ranging from the development of mastocytomas at injections sites to severe anaphylaxis [46,47,48,49,50].

Reactions appear to be transient without recurrence on booster administrations [45,50].

Combination vaccines seem to increase risk for reactions more than single shots, since most reactions were described following hexavalent formulations. Consequently, it is safer to consider single vaccine injections in substitution of the combined ones [45].

Physicians should adequately inform parents and children, reassuring and educating them about the management of adverse reactions [51]. Vaccinations are still recommended in children with mastocytosis since benefits outweigh potential disadvantages. Nevertheless, it is reasonable to be cautious and administer vaccinations in a clinical setting provided with adequate drugs, including epinephrine, putting children on observation for a prolonged time (30 min according to Zanoni et al. [52], 2 h according to Parente et al. [50]) following the administration.

## 9. Pharmacological Treatment

The treatment of mild clinical manifestations is largely symptomatic and consists of:

(1) H1-histamine receptor blockers, for general symptoms (such as Cetirizine, Levocetirizine, Loratadine, Desloratadine, Fexofenadine);
(2) H2-histamine receptor blockers, mainly for gastrointestinal symptoms (Ranitidine, Cimetidine);
(3) Leukotriene receptor blockers (such as Montelukast, Zafirlukast and Zileuton); and
(4) Mast cell stabilizers (Cromolyn, Nedocromil).

### 9.1. H1/H2-Histamine Receptor Blockers

Though widely used, a recent systematic review found that little evidence supports this habitude. Authors found only one well-designed randomized controlled trial (RCT), which demonstrated rupatadine to be superior to placebo with a globally significant improvement of clinical conditions of adults suffering from MCAS symptoms [53].

### 9.2. Leukotriene Receptor Blockers

The rational for their use in MCs’ disorders lays on the massive release of leukotrienes by MCs’ degranulation. Nevertheless, no strong evidence has been found yet about their effectiveness [38].

### 9.3. Mast Cell Stabilizers

Instead, mast cell stabilizers, especially cromolyn sodium, have shown efficacy in different RCTs [38].

## 10. Other Therapeutic Options

In a previously cited retrospective study from Ravi et al. [22], the use of acetylsalicylic acid for MCAS was mentioned. The rational lays on the production of PGD2 by MCs, directly depending on prostaglandin–endoperoxide synthase, more commonly known as cyclooxygenase (COX), whose function is blocked by acetylsalicylic acid. Most patients treated with acetylsalicylic acid and with elevated 9a-11b-PGF2 urinary levels responded with both an improvement of symptoms and normalization of 9a-11b-PGF2 levels [22].

Anti-Immunoglobulin (-Ig)E recombinant humanized monoclonal antibody Omalizumab has been tested and proven to be effective in reducing symptoms in primary, secondary, and idiopathic MCAS other than in pediatric diffuse CM [41].

Patients only suffering from CM may benefit from topical emollients, topical calcineurin inhibitors, or phototherapy [38].

### 10.1. Tyrosine Kinase Inhibitors

The theoretical possibility that a tyrosine kinase inhibitor, such as Imatinib, could block the pathological reaction has been proposed. Unfortunately, trials conducted on patients affected by SM with c-Kit D816V mutation with tyrosine kinase inhibitors showed that the mutation itself confers resistance to Imatinib, such as to other tyrosine kinase inhibitors (Dasatinib, Masitinib, Nilotinib). This is likely due to a conformational change of the activation loop, interfering with the binding of imatinib to the receptor [54].

A multicenter experiment about the role of Imatinib in the treatment of “advanced MC disease” reported a 29% efficacy only in patients without D816V c-kit mutation [55].

Nevertheless, Imatinib has been found to be useful in SM with different c-Kit mutations involving transmembrane receptors, mainly F522C and K509I [31,37]. To the best of our knowledge, a trial with Imatinibin MCAS has never been published.

Ustun et al. recommended to avoid the use of Imatinib for SM in pediatric patients because of the self-limiting feature of some of the childhood-onset mastocytosis and because of the lack of knowledge about long-term adverse effects of imatinib in children [54].

### 10.2. Midostaurin

A promising therapeutic agent is represented by midostaurin, an N-benzoyl derivative of staurosporine, initially developed as a protein kinase C (PKC)-targeting drug that, later, proved to be a multitargeted kinase inhibitor. In particular, midostaurin appears to be very promising since it exhibits an ability to suppress D816V c-Kit mutated clonal cells and, at the same time, it showed the ability to inhibit IgE-dependent mediator release in MCs and basophils [56].

The cornerstone of midostaurin’s use in MC disorders was the huge multicenter, open-label, phase II (single-arm) trial by Gotlib et al. [57] conducted on patients with advanced SM. Patients showed a 60% response rate even though the drug was not globally well tolerated by patients since 52% were forced to temporarily discontinue therapy [57]. Data of its use in children are lacking.

### 10.3. Cytoreductive Therapies

Interferon alpha-2b was studied on patients suffering from D816V c-Kit mutated SM not responding to less invasive therapies, combined with prednisolone by Hauswirth et al. [58]. Despite the small number of patients included in the study (five, all suffering from ASM), the authors obtained a good response in 3/5 (60%) patients with complete or partial resolution of symptoms and stabilized the disease in 1/5 (20%). One of the included patients (20%), unluckily, decided to interrupt the therapy because of side effects [58].

The 2-chloro-2′-deoxyadenosine, or Cladribine, is an antimetabolite, currently approved to treat Hairy Cell Leukemia and Multiple Sclerosis [59].

Ever since 2001, it has been experimented in SM patients, leading to response rates > 50% in SM [42].

## 11. Conclusions

MCAS is a highly complex disease with associated disorders ranging from relatively common IgE-mediated disease to less frequent conditions such as mastocytosis or monoclonal MC activation disorder. The diagnostic criteria established by an international consensus should always be applied [1]. Serum total tryptase is considered the preferred enzyme for diagnosing MCAS, but no consensus was found on different biomarkers. The level of the tryptase should be higher than 20% plus 2 ng/mL.

Based on the underlying condition, patients with MCAS should then be further classified into primary/clonal MCAS, secondary MCAS, or idiopathic MCAS.

No definitive treatment is yet available for MCAS, so its management must be multidisciplinary, integrated, and aimed primarily at avoiding triggers and the risk of symptom recurrence.

Clinical practitioners’ training and well-designed and thought-out prospective clinical research protocols are needed for improved recognition and knowledge of this syndrome and treatments.

## Figures and Tables

**Table 1:** MCAS (Mastcell activation syndrome) recognizes three different subtypes.

Definition	Main Features
**Primary/Clonal MCAS**	D816V detectable in C-Kit gene with/without CD25 expression in clonal MCs
**Secondary MCAS**	Detectable conditions such as atopy, autoimmune diseases, bacterial infections, adverse drug reactions without mutations in CD25 or C-Kit
**Idiopathic MCAS**	Diagnosis of exclusion

**Table 2:** World Health Organization (WHO) classification of mastocytosis (adapted from Pardanani [31]).

1. Cutaneous mastocytosis (CM)
2. Indolent systemic mastocytosis (ISM)
3. Smoldering systemic mastocytosis (SSM)
4. Systemic mastocytosis with an associated hematological neoplasm (SM-AHN)
5. Aggressive systemic mastocytosis (ASM)
6. Mast cell leukemia (MCL)
7. Mast cell sarcoma (MCS)

**Table 3:** SM (Systemic Mastocytosis) diagnostic criteria (Adapted from Valent et al. [37]).

Major Criterion	Multifocal Dense Infiltrates of MCs (≥15 MCs in Aggregates) in Bone Marrow Biopsies and/or in Extracutaneous Organs
Minor criteria	a. >25% of all MCs are atypical cells (type I or type II) on BM or are spindle-shaped in MC infiltrates detected on sections of visceral organs
	b. D816V C-Kit mutation in cells from bone marrow, blood or extracutaneous organs
	c. CD2 and/or CD25 expressed by cells from bone marrow, blood or extracutaneous organs
	d. Baseline serum tryptase level > 20 ng/mL (Warning: this criterion must not be counted if a myeloid neoplasm is present)
Diagnosis can be made if: ● 1 Major criteria + at least 1 minor criteria are present; ● No major criteria but at least 3 minor criteria are present.	

**Table 4:** Differential diagnoses of primary MC activation conditions (adapted from Weiler and Caimmi [25,41]).

Cutaneous	Acquired angioedema
	Hereditary angioedema
	Idiopathic flushing
	Erythema nodosum
	Atopic dermatitis
	Allergy contact dermatitis
Autoimmune disorders	Vasculitis
	Systemic lupus erythematosus (SLE)
Endocrine	Thyroid dysfunction
	Adrenal gland dysfunction
Cardiovascular	Myocardial Infarction
	Myocarditis
	Hypovolemic shock
Gastrointestinal	Inflammatory bowel disease
	Irritable bowel syndrome
Neoplasms	VIPoma
	Carcinoid tumours
	Pheochromocytoma
	Medullary thyroid carcinoma
Neurological	Stroke
	Multiple Sclerosis
Miscellanea	Serotonin syndrome
	Scombroid syndrome
	Panic attacks
	Toxic or allergic reactions

**Table 5:** Factors that trigger degranulation of MCs and recommended premedication regimens (Adapted from Castells and Butterfield [42]).

**A. Avoidance of Triggers: Patient Specific**
**Specific foods, medications (NSAIDs, vancomycin, quinolones), environmental allergens, and general triggers (stress, lack of sleep, emotions)**
**Physical triggers (exercise, rubbing, pressure)**
**Changes in temperature (heat, cold)**
Extreme temperatures
Dryness of skin
**B. Premedications Recommended for Surgery, Invasive Procedures (Endoscopy, Colonoscopy, Others), radiological Procedures with Contrast Dyes, Dental Procedures, and Vaccinations: 12 and 1 h**
**Antihistamine receptors H1 and H2**
Leukotriene blocker
Steroid
NSAID, nonsteroidal anti-inflammatory drug

## Data Availability

The study not report any data.
